# Altered gut metabolome contributes to depression-like behaviors in rats exposed to chronic unpredictable mild stress

**DOI:** 10.1038/s41398-019-0391-z

**Published:** 2019-01-29

**Authors:** Li Jianguo, Jia Xueyang, Wang Cui, Wu Changxin, Qin Xuemei

**Affiliations:** 1 Laboratory of Microbiome and Health, Institutes of Biomedical Sciences, Shanxi University, Taiyuan, 030006 China; 2 Key Laboratory of Chemical Biology and Molecular Engineering of Ministry of Education, Shanxi University, Taiyuan, 030006 China; 3 Modern Research Center for Traditional Chinese Medicine, Shanxi University, Taiyuan, 030006 China

**Keywords:** Depression, Physiology

## Abstract

The gut microbiota has been increasingly correlated with depressive disorder. It was recently shown that the transplantation of the gut microbiota from depressed patients to animals can produce depressive-like behaviors, suggesting that the gut microbiota plays a causal role in the development of depression. In addition, metabolic disorder, which is strongly associated with depression, is exacerbated by changes in the composition of the gut microbiota and is alleviated by treatment with antidepressants. However, the key players and pathways that link the gut microbiota to the pathogenesis of depression remain largely unknown. To evaluate the relationships between depression and metabolic disorders in feces and plasma, we monitored changes in fecal and plasma metabolomes during the development of depressive-like behaviors in rats exposed to chronic unpredictable mild stress (CUMS). In these animals, the fecal metabolome was altered first and subjected to changes in the plasma metabolome. Changes in the abundance of fecal metabolites were associated with depressive-like behaviors and with altered levels of neurotransmitters in the hippocampus. Furthermore, the analysis of the fecal metabolome and the fecal microbiota in CUMS rats demonstrated consistent changes in the levels of several amino acids, including L-threonine, isoleucine, alanine, serine, tyrosine, and oxidized proline. Finally, we observed significant correlations between these amino acids and the altered fecal microbiota. The results of this study suggest that changes in amino acid metabolism by the gut microbiota contribute to changes in circulating amino acids and are associated with the behavior indices of depression.

## Introduction

Major depressive disorder (MDD) is a widespread mood disorder that has significant adverse effects on personal health and results in staggering medical costs^1^. Despite a battery of psychological and pharmacological treatments for depression, only 74% of patients with MDD show improvement^2^. Thus, there is an urgent need to develop more efficacious therapies and to identify the additional causal factors of depression^3,4^.

Although MDD is primarily a psychological condition, the overall physiology of depression is embedded in the central nervous system^5^. The symptoms of depression are far-reaching, including weight loss^6^, sleeping difficulties^7^, and psychomotor agitation^8^. A more comprehensive understanding of the pathophysiology of depression will, therefore, require a multifaceted investigation of the physiological factors that contribute to depression^9,10^. Depression has been associated with changes in several physiological pathways, including adipose-derived hormones^11^, insulin signaling^12^, inflammatory cytokines^10^, the hypothalamic–pituitary–adrenal axis^1,13^, and oxidative stress pathways^14^. In addition, metabolic disorder has recently been recognized as a characteristic of depression^15,16^ and as a potential target for antidepressant therapies^17^.

The gut microbiota has been strongly associated with depression^18,19^, and this association has been attributed to the bi-directional communication between the gut and the brain^4,20,21^. Decreases in the diversity and richness of the gut microbiota are associated with depression^22^. Moreover, fecal transplantation from patients with MDD replicates depressive symptoms in recipient rodents^23^. Thus, changes in the gut microbiota are likely to contribute to the pathogenesis of depression^24^. The adoption of depressive behaviors also results in reduced richness and diversity of the gut microbiota, suggesting that the gut microbiota and depression can impact one another^19^. However, despite this evidence, the key players in this process and the precise molecular mechanisms by which the gut microbiota and depression impact one another are not yet clear^4,19^.

Pathophysiological and pharmacological studies demonstrate that metabolic disorders occur in the plasma/serum and in the central nervous system of depressed patients^25,26^ and in animal models with depressive-like behaviors^27,28^. These changes can be rebalanced by the treatment with antidepressants^29,30^. For example, plasma alpha-aminobutyric acid has been shown to be a potential biomarker of MDD and a predictor of therapeutic response in MDD^31^. This close relationship between the gut microbiota and the pathomechanisms of depression highlights the potential key role of gut microbiota in depression. Thus, in addition to understanding the molecular mechanisms by which the gut microbiota contributes to changes in the metabolome in depression, it is essential to determine the timing of these changes. This timing will be crucial in understanding how the two disorders influence one another^32^.

To address these key questions, we performed a temporal dynamic gas chromatography–mass spectrometry (GC–MS) analysis of the fecal and plasma metabolomes during the development of depressive-like behaviors in rats. Animal models of depression have substantial advantages because they can establish relationships between a wide range of possible causal factors^33–35^. For these analyses, we induced depressive-like behaviors by exposing rats to chronic unpredictable mild stress (CUMS), which is widely accepted to most closely mimic the social stressors suffered by patients with MDD^36–38^. Using this system, we determined the precise timing of changes in the fecal and plasma metabolomes and explored how the gut microbiota contributes to changes in the plasma metabolome and other depressive-like symptoms.

## Materials and methods

### Animals and reagents

Eight-week-old male Sprague–Dawley rats with an average body weight of 200 g ( ± 10 g) were purchased from Beijing Vital River Laboratories Co. (SCXK (Jing) 2011–2012). To ensure adequate statistical power, six rats were randomly enrolled in each group by a random number generator implemented in SPSS 22.0 (Chicago, USA). The rats were housed with free access to food and water and kept at a controlled room temperature of 25 °C ( ± 1 °C), humidity (45 ± 15%), and light (lights on at 8:00 a.m., 12-h day/night switch). Newly purchased rats were acclimatized to their new environment for 1 week prior to the start of experiments. All experimental procedures were approved by the Committee on Animal Research and Ethics of Shanxi University.

Neurotransmitter standards, including 5-hydroxytryptamine (5-HT), norepinephrine (NE), tryptophan (TRP), gamma-aminobutyric acid (GABA), 3,4-dihydroxyphenylacetic acid (DOPAC), kynurenine (KYN), 3-methoxytyramine (3-MT), 5-hydroxyindole acetic acid (5-HIAA), 3-hydroxykynurenine (3-HK), 3-hydroxyanthranilic acid (3-HAA), and dopamine (DA) were purchased from Sigma-Aldrich (St. Louis, MO, USA). Homovanillic acid and the derivatization reagent dansyl chloride were purchased from Tokyo Chemical Industry Co. Ltd. (Tokyo, Japan). Formic acid, acetone, methanol, and acetonitrile (LC-MS grade) were obtained from Merck (Darmstadt, Germany). All solvents were of High Performance Liquid Chromatography (HPLC) grade or above.

### CUMS modeling

To determine the timing of metabolome changes in the feces and plasma of depressive animals, CUMS modeling was performed according to the protocol described previously^39^. Briefly, rats were housed individually and subjected to 2–4 of the following stressors every day in a random order for 4 weeks: foot shock for 2 min, swimming in 4 °C water for 5 min, tail clamp for 2 min, subject to noise for 3 h, food deprivation for 24 h, water deprivation for 24 h, and subject to room temperature at 45 °C for 5 min. Fecal samples were collected using metabolic cages every week. Orbital blood samples were obtained every week, and femoral artery blood samples were collected after scarification with ethyl carbamate anesthesia. Ethylenediaminetetraacetic acid-anticoagulated blood was centrifuged at 4 °C, 3000 rpm for 15 min, and the supernatants were stored at −80 °C.

### Body weight measurement

The body weight of each rat was measured at 8 a.m. on days 0, 7, 14, 21, and 28 of the CUMS modeling process.

### Sucrose preference test

One bottle of tap water and another bottle of 1% sucrose solution were provided to each rat for 4 h, and the consumption of sucrose and water was recorded. The sucrose preference rate was calculated as sucrose consumption (g)/(sucrose consumption (g) + water consumption (g)). To avoid neophobia, exposure to 1% sucrose solution for 24 h was performed before sucrose preference test.

### Open-field test

To evaluate the changes in learning and memory of rats exposed to CUMS, open-field tests (OFTs) were carried out once a week. Each rat was monitored for 5 min, and grooming time, immobility time, rearing, and crossing counts were recorded.

### GC–MS

GC–MS analysis of fecal and plasma samples was conducted as previously described^39^. Briefly, for fecal samples, 200 mg of dried feces was homogenized in 500 μl water and centrifuged at 13,000 rpm for 10 min. The supernatant was then transferred to 400 μl of acetonitrile for protein precipitation. For plasma samples, 500 μl of the plasma was added to 400 μl of acetonitrile for protein precipitation. A second centrifugation similar to the above was performed, and the supernatant was thoroughly dried in a nitrogen concentrator and re-suspended in 30 μl of pyridine-methoxy amino acid salt solution (15 mg/ml). The solution was subsequently incubated at 70 °C for 1 h, and 50 μl of N,O-bis (trimethylsilyl) tri-fluoroacetamide (including 1% trimethylchlorosilane) was added before another incubation at 40 °C for 1.5 h. One microliter of each analyte was injected in a (10:1) split mode into a trace gas chromatograph coupled with a Polyris Q Ion Trap mass spectrometer (Thermo Fisher Scientific, MA, USA). Ethyl Chloroformate (ECF) derivatives were separated with a DB-5MS capillary column (30 m × 250 μm i.d., 0.25 μm film thickness; Agilent J & W Scientific, CA, USA). Helium was used as the carrier gas at a constant flow rate of 1.0 ml/min. The oven temperature for GC–MS was first held at 80 °C for 3 min, ramped to 140 °C at a speed of 7 °C/min, held at 140 °C for 4 min, ramped to 180 °C at a speed of 4 °C /min, held at 180 °C for 6 min, then ramped to 280 °C at a speed of 5 °C /min and held at 280 °C for 2 min.

### Neurotransmitter quantitation

Ultra-high performance liquid chromatography-Electrospray Ionization -Tandem Mass Spectrometry (UHPLC-ESI-MS/MS) quantitation of 12 neurotransmitters in the hippocampus was carried out according to the previously reported methods with some modifications^40,41^. Briefly, 30–50 mg of rat hippocampus sample was homogenized and precipitated with methanol. The supernatant was completely dried with a nitrogen concentrator and reconstituted with the initial mobile phase of UHPLC. UHPLC-ESI-MS/MS was run on a Thermo Scientific Dionex Ultimate 3000 RSLC system, combined with a Thermo Q Exactive Orbitrap mass spectrometer. The analytes were separated with a Thermo Hypersill GOLD (2.1 × 100 mm, 1.7 μm) column. The mobile phase consisting of phases A (water:formic acid (99.9:0.1, v/v)) and B (acetonitrile:formic acid (99.9:0.1, v/v)) was used with a gradient elution at a flow rate of 0.3 ml/min: linear increase from 0% B to 20% B in 3 min; hold at 60% B for 3 min; linear increase from 60% B to 80% B in 4 min; linear increase from 80% B to 95% B in 3 min; hold at 95% B for 4 min. ESI-MS/MS conditions were set as follows: gas temperature, 350 °C; gas flow, 46 ml/min; capillary voltage, 3000 V; and nebulizer pressure, 35 ps. MS acquisitions were performed in the parallel reaction monitoring mode.

### 16s rDNA amplicon sequencing

Total bacterial DNA was extracted from fecal samples using the Fast DNA SPIN kit for feces (MPBIO, CA, USA) according to the manufacturer’s instructions. DNA integrity tests were performed by agarose gel electrophoresis. The V4 region of 16 s rDNA was targeted and polymerase chain reaction (PCR) amplified using the primer pairs 515F/806R (515F: 5′-TGTGCCAGCMGC CGCGGTAA-3′; 806R: 5′GGACTACHVGGGTWTCTAAT-3′). The PCR cycling conditions were as follows: 98 °C for 30 s, followed by 35 cycles of 98 °C for 5 s, 56 °C for 20 s, and 70 °C for 20 s. The PCR amplicons were purified with AmpureXp beads (AGENCOURT) to remove nonspecific products. PCR products were checked using a Bioanalyzer DNA 1000 chip (Agilent Technologies, CA, USA), and libraries were qualified and pair-end sequenced using the PE300 sequencing strategy provided by the Illumina MiSeq system.

### Data acquisition for GC–MS and neurotransmitter quantitation

For GC–MS, mass data were collected in full scan mode from 50 to 650 m/z. Compounds were identified by comparison of mass spectra with those in the National Institute of Standards and Technology library (version 2.0). The Human Metabolome Database (http://www.hmdb.ca) was used for further verification. The identified metabolites were validated with commercially available standards. The GC–MS-generated raw result files were converted to Net-CDF format and then processed by XCMS using default settings.

To quantify the neurotransmitters, the calibration curve for each analyte was obtained by linear regression analysis with 1/*x*^2^ weighting factor, which contained 10 points covering a linear range of 0.02–20 ng. Data acquisition and analysis were performed with Thermo Xcalibur 2.2 software.

### Metabolome analysis

To eliminate the differences and to increase the orthogonality of metabolomic features, the metabolomic datasets were first normalized to constant sum and scaled with Pareto scaling using SIMCA-P 13.0 (Umetrics AB, Umea, Sweden). Principal component analysis was performed to explore the natural separation between the study groups. Orthogonal projection to latent structure discriminate analysis (OPLS-DA) was used to investigate the difference between groups by incorporating known classification information. Metabolites with variable importance for the projection values greater than 1 in the established OPLS-DA model, and false discovery rate-adjusted *P* values *<* 0.05 in an independent-samples *t* test were designated as differential metabolites contributing to the separation of the study groups. Metabolic pathway enrichment of the between-group differential metabolites was performed by the MetaboAnalyst web portal (http://www.metaboanalyst.ca).

### Metagenome analysis

A set of parameters were selected to filter the raw reads of 16s rDNA sequencing data, including a minimum sequence length of 150 bp, a minimum Q-score of 20, a maximum number of consecutive N of 0, and a maximum of three consecutive low-quality base calls allowed before truncating. The number of filtered sequences per sample ranged from 47,243 to 59,454, with a mean of 52,470. FLASH v1.2.7 was used to assemble the pair-end sequencing reads. Operational taxonomic units (OTUs) were picked using UCLUST in QIIME pipeline (v1.8.0) against the GreenGenes database (the May 2013 version, http://greengenes.secondgenome.com) at 97% identity. OTUs with a relative abundance lower than 0.001% of the total OTUs were removed^42^, leaving OTUs per sample ranging from 367 to 536 at the genus level. LEfSe^43^ was used to assess biomarkers (linear discriminant analysis score > 3.0) from the relative abundance of bacterial taxonomy. An adjusted *P* value *<* 0.05 was defined as statistically significant. The metabolic potential of the altered microbiota was predicted by Phylogenetic Investigation of Communities by Reconstruction of Unobserved STates (PICRUSt) analysis^44^.

### PERMANOVA analysis

Permutational multivariate analysis of variance (PERMANOVA) was performed on the metabolite-abundance profiles to assess the effect of each behavior index using Bray–Curtis distance and 9999 permutations in R (v3.5.0, vegan package). Behavior indices with adjusted *P* values *<* 0.05 were defined as being significantly associated with metabolites.

### Co-inertia analysis

Co-inertia analysis (CIA) was performed on metabolomic abundance profiles of feces and plasma to assess the relationships between the fecal metabolome and the plasma metabolome. The CIA plot was generated by R software (v3.5.0, package vegan) using default parameters.

### Canonical correspondence analysis

Canonical correspondence analysis (CCA) was performed to assess the effect of altered fecal metabolites on the separation between CUMS-enriched and healthy control-enriched plasma metabolites. The differential enrichments of altered plasma metabolites were determined by odds ratio analysis. The CCA plot was generated by R software (v3.5.0, package vegan) with default parameters.

### Procrustes analysis

Procrustes analysis (PA) was performed to assess the similarity between the altered fecal metabolome and the plasma metabolome. PA was based on the superimposition of principal coordinates constructed from the distance matrices calculated from the square root of the Jensen–Shannon divergence. The significance of PA was determined by 10,000 Monte Carlo permutations in QIIME.

### Correlation analysis

Spearman correlations among the altered microbiota genera, the altered metabolites, depressive-like behavior indices, and neurotransmitters in hippocampus were calculated and plotted in R (v3.5.0, package corrplot). Correlations with absolute coefficient values > 0.6 and adjusted *P* values < 0.05 were defined as statistically significant.

### Statistical analysis

Data for spectrometry profiling, behavior indexing, and neurotransmitter quantitation are expressed as the mean ± SEM. Statistical analyses were performed with two-tailed Student’s *t tests* in SPSS 22.0. *P* values were adjusted with false discovery rate correction in *R* (v3.5.0, package p.adjust), and adjusted *P* values *<* 0.05 were defined as statistically significant. If a between-group variation was statistically significant, one-way analysis of variance was performed to estimate the within-group variation.

## Results

### Depressive-like phenotypes arise at different times during exposure to CUMS

**Fig. 1:**
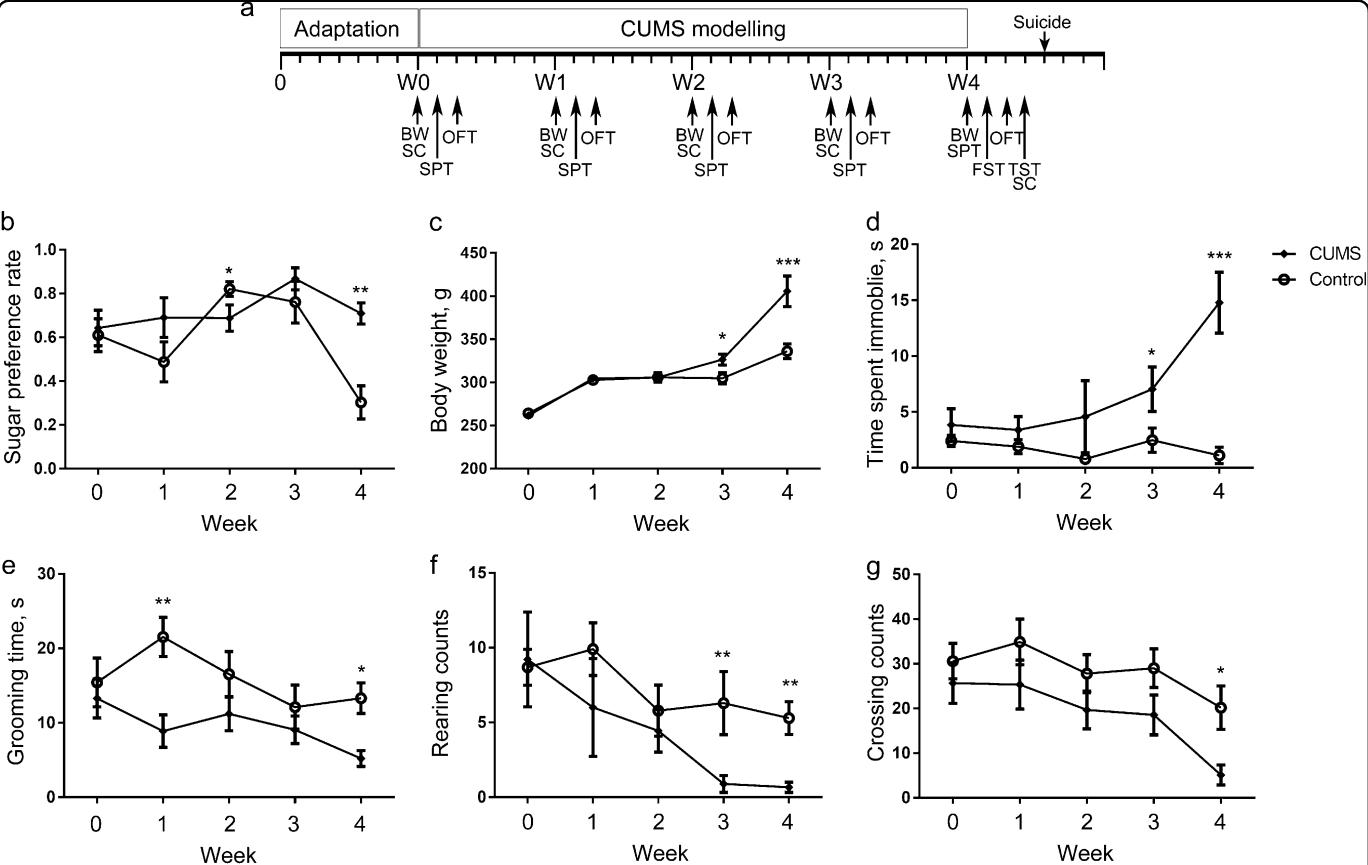
Experimental procedures and temporal dynamics of behavior indices. **a** Animals were exposed to CUMS as described in the materials and methods section for 4 weeks (W1–W4) after the adaptation to a new environment for 1 week (W0). Fecal and plasma samples were collected weekly. Sugar preference rate (**b**), body weight (**c**), and behavior indices of open-field test (**d**–**g**) were recorded every week. To avoid the possible interference with CUMS modeling, tail suspension test and forced swimming test were performed not weekly but after a 4-week CUMS exposure. Student’s *t* tests were applied to calculate between-group statistical significance. *P *<* 0.05, **P *<* 0.01, and ***P *<* 0.001. BW, body weight; CUMS, chronic unpredictable mild stress; FST, forced swimming test; OFT, open-field test; SC, sample collection; SPT, sucrose preference test; TST, tail suspension test

CUMS is a widely accepted animal model of depressive-like behaviors that mimics the social stressors suffered by human beings^9,45^. While much attention has been paid to the terminal state of CUMS, comparatively little is known about the timing of symptom origin^28,36–39^. To investigate the role of the gut microbiota in the development of depressive-like symptoms, we monitored the temporal dynamics of experimental phenotypes during the CUMS modeling process (Fig. 1a). After the final week of CUMS exposure, we observed a notable decrease in sugar preference rate (Supplementary Fig. 1a) and significant increases in the time spent immobile in OFT (Supplementary Fig. 1b), forced swimming test (Supplementary Fig. 1c), and tail suspension test (Supplementary Fig. 1d). We also observed clear separations of metabolome in the hippocampus (Supplementary Fig. 2a) and prefrontal cortex (Supplementary Fig. 2b) at the same time point. These results suggested that our CUMS modeling was successful and could be used to study the contributions of gut microbiota to the development of depressive-like behaviors.

Although we observed depressive-like phenotypes after a 4-week CUMS modeling, the temporal dynamics of each experimental phenotype differed. For example, we observed significant differences in body weight (Fig. 1c), two OFT indices (time spent immobile (Fig. 1d)), and rearing counts (Fig. 1f) starting in the 3rd week of exposure to CUMS. We did not observe significant changes in sucrose preference rate (Fig. 1b), grooming time (Fig. 1e) or crossing counts (Fig. 1g) of OFT until the last week of CUMS modeling, although an isolated notable change was evident in grooming time (Fig. 1e) at the 1st week. These results suggest that depressive-like behaviors develop at different time points in CUMS rats.

### Fecal metabolome disorder arose before plasma metabolome disorder during exposure to CUMS

**Fig. 2:**
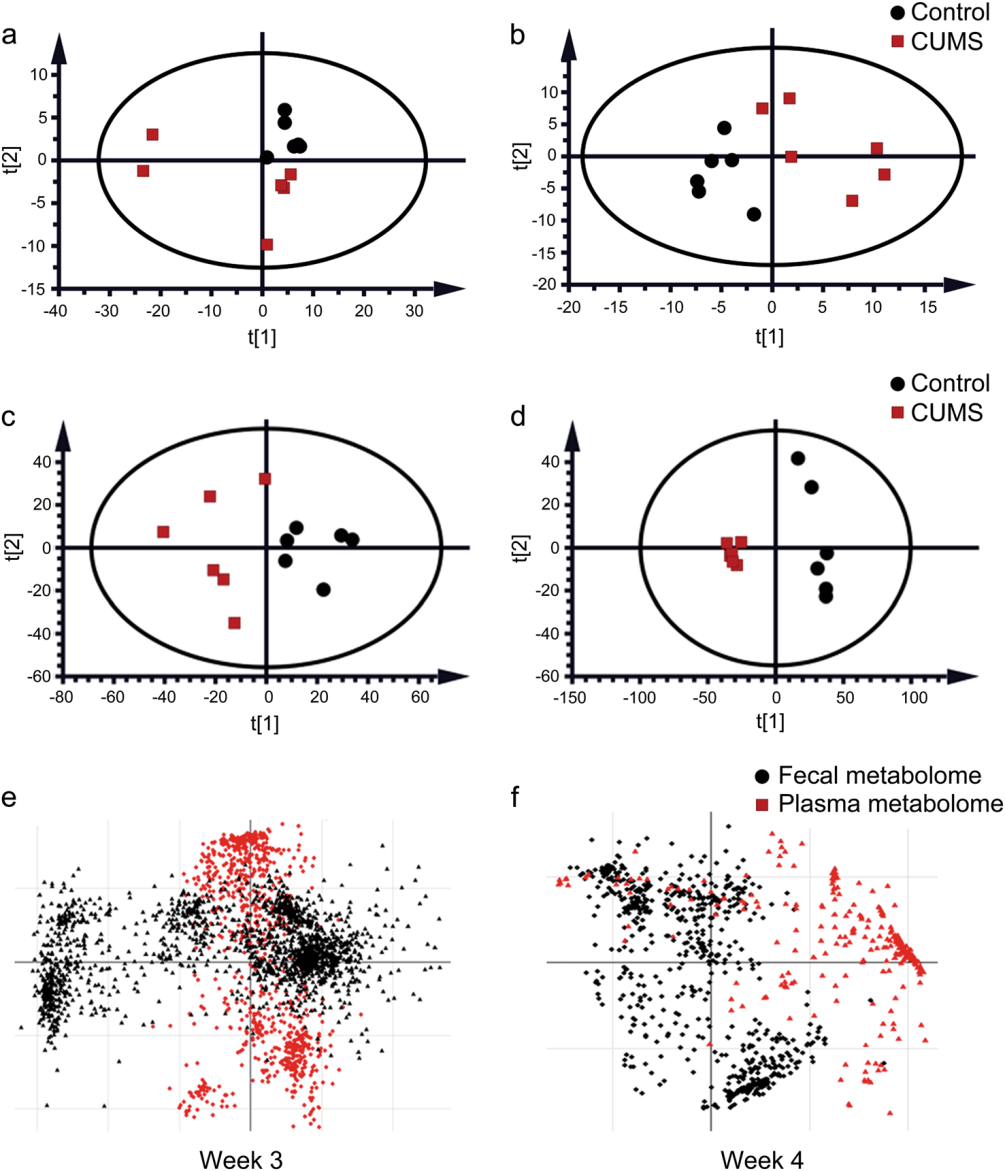
Principal component analysis and co-inertia analysis of the plasma and fecal metabolomes in CUMS rats and healthy controls. Principal component analyses (PCAs) were performed to compare the between-group variations in metabolomic data from plasma (**a**–**b**) and fecal (**c**–**d**) samples obtained at the 3rd and 4th week (W3–W4) of CUMS exposure. The first two axes (PC1 and PC2) were selected for the score plots. The red dots represent the CUMS group, and the black dots represent the healthy control group. Co-inertia analyses (CIAs) were performed with fecal and plasma metabolome data obtained from the 3rd and 4th week (W3–W4) of CUMS exposure (**e**, **f**). The dots represent metabolites or unidentified features of the metabolomic data, black dots represent features of the fecal metabolome, and red dots represent features of the plasma metabolome. The full results of PCAs and CIAs for data from all of the five time points (W0–W4) of CUMS exposure were listed in Supplementary Fig. 3

The circulating metabolome has been reported to be a key source of biomarkers of depression^46,47^, and the gut microbiota/metabolome has been suggested as a critical influencing factor^48,49^. To investigate the reasons that underlie the asynchronous development of depressive-like behaviors, we analyzed the plasma (Fig. 2 a, b, Supplementary Fig. 3 a1–a5) and fecal metabolomes (Fig. 2 c, d, Supplementary Fig. 3 b1–b5) from the five time points of the CUMS modeling process (Fig. 1a) using GC–MS. The results of principal component analysis demonstrated clear differences between the fecal metabolome of CUMS rats and that of healthy controls beginning in the 3rd week of CUMS exposure (Fig. 2 c, d), but no significant difference in the plasma metabolome occurred until the 4th week of CUMS exposure (Fig. 2 a, b). These findings demonstrate that changes in the fecal metabolome arise before changes in the plasma metabolome in CUMS rats during the development of depressive-like behaviors, suggesting that the fecal metabolome has an effect on the plasma metabolome.

### Changes in the fecal metabolome are associated with depressive-like behaviors

Behavior indices are key indicators of depressive-like symptoms in CUMS rats^34^. Because changes in the fecal metabolome (Fig. 2 c, d, Supplementary Fig. 3 b1–b5) coincided with the initiation of depressive-like symptoms (Fig. 1 b–g), we investigated a potential correlation between depressive-like symptoms and the altered fecal metabolome. Based on PERMANOVA, sucrose preference rate, body weight, and all indices of OFT were significantly correlated with the altered fecal metabolome (adjusted *P* < 0.05; Table 1) in the 4th week of the CUMS modeling, suggesting that changes in the fecal metabolome contribute to depressive-like behaviors. In addition, we observed significant correlations between the altered fecal microbiota in the 4th week of CUMS exposure and the behavior indices (adjusted *P* value < 0.05; Table 2). These results suggest that the altered gut microbiota affects the depressive-like symptoms of CUMS rats, possibly through the altered gut metabolome.

**Table 1:** PERMANOVA ranking the association between the altered fecal metabolites and behavior indices/neurotransmitters in the hippocampus

Phenotype	Degree of freedom	*R*^2^	*P* value	Adjusted *P* value
Body weight	1	0.06270267	1.10E-03	2.38E-03
Sucrose preference rate	1	0.00683232	8.00E-03	1.39E-02
Grooming time	1	0.01321207	4.08E-02	6.63E-02
Immobility time	1	0.01229415	6.55E-02	1.00E-01
Rearing counts	1	0.06455333	1.10E-03	2.38E-03
Crossing counts	1	0.01299891	3.17E-01	4.58E-01
3-MT	1	0.0517155	1.10E-03	2.38E-03
NE	1	0.03423785	1.10E-03	2.38E-03
5-HT	1	0.03661607	1.10E-03	2.38E-03
DOPAC	1	0.02254633	7.50E-03	1.39E-02
HVA	1	0.06469889	1.10E-03	2.38E-03
KYN	1	0.05594343	1.10E-03	2.38E-03
TRP	1	0.04790959	1.10E-03	2.38E-03
3-HK	1	0.05785465	1.10E-03	2.38E-03
GABA	1	0.02100625	1.30E-03	2.60E-03
5-HIAA	1	0.03044673	1.10E-03	2.38E-03
3-HIAA	1	0.05556307	1.10E-03	2.38E-03
DA	1	0.0444571	1.10E-03	2.38E-03

*PERMANOVA* permutational multivariate analysis of variance

**Table 2:** PERMANOVA ranking the association between fecal microbiota genera and behavior indices/neurotransmitters in the hippocampus

Phenotype	Degree of freedom	*R*^2^	*P* value	Adjusted *P* value
Body weight	1	0.0441	1.00E-04	2.36E-04
Sucrose preference rate	1	0.0318	1.00E-04	2.36E-04
Grooming time	1	0.0432	1.00E-04	2.36E-04
Immobility time	1	0.0402	1.00E-04	2.36E-04
Rearing counts	1	0.0377	1.00E-04	2.36E-04
Crossing counts	1	0.0271	2.00E-04	4.33E-04
3-MT	1	0.0505	1.00E-04	2.36E-04
NE	1	0.0167	5.64E-01	8.95E-01
5-HT	1	0.0091	5.85E-01	8.95E-01
DOPAC	1	0.0476	1.00E-04	2.36E-04
HVA	1	0.0523	1.00E-04	2.36E-04
KYN	1	0.0133	6.38E-02	1.11E-01
TRP	1	0.0394	1.00E-04	2.36E-04
GABA	1	0.0170	8.80E-03	1.63E-02
3-HK	1	0.0098	7.89E-01	1
5-HIAA	1	0.0538	1.00E-04	2.36E-04
3-HIAA	1	0.0457	1.00E-04	2.36E-04
DA	1	0.0183	4.30E-03	8.60E-03

*PERMANOVA* permutational multivariate analysis of variance

### Changes in the fecal metabolic profile are associated with changes in the plasma metabolome of CUMS rats

Changes in the plasma metabolome have been reported to be correlated with depression in both humans^25^ and animal models with depressive-like behaviors^21^. To study the relationships between the fecal and plasma metabolomes during the development of depressive-like behaviors, CIA was performed with metabolome data obtained from each time point during CUMS modeling process (Fig. 2 e, f, Supplementary Fig. 3 c1–c5). The fecal and plasma metabolomes were tightly clustered at weeks 0 and 1 of CUMS modeling (Supplementary Fig. 3 c1, c2), suggesting little correlation between each other. In later time points, closer to the onset of depressive-like behaviors, we observed a separation trend between the metabolome of feces and plasma (Fig. 2e, Supplementary Fig. 3 c3, c4). A clear separation appeared at the 4th week of CUMS modeling (Fig. 2f, Supplementary Fig. 3 c5), demonstrating a correlation between the fecal and plasma metabolomes in CUMS rats.

**Fig. 3:**
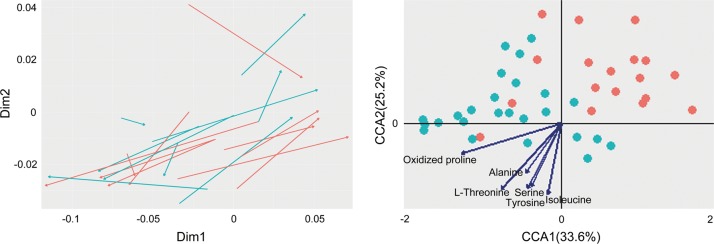
Procrustes analysis and canonical correspondence analysis between fecal and plasma metabolome. Procrustes analysis (PA) was performed based on Bray–Curtis dissimilarity to quantify the dissimilarity of the relative abundances of the differential fecal and plasma metabolites between the CUMS group and the healthy control group (**a**). Red edges: fecal metabolome; green edges: plasma metabolome. **b** Correspondence analysis (CCA) was performed to assess the contributions of the differential fecal metabolites to the separation of plasma metabolome between the CUMS group and the healthy control group. Green dots: control-enriched GC–MS spectral features of the plasma metabolome; red dots: CUMS-enriched GC–MS spectral features of the plasma metabolome. Only correlations with coefficient > 0.6 and *P* < 0.05 in the corresponding significance test are shown. CUMS, chronic unpredictable mild stress; GC–MS, gas chromatography–mass spectrometry

To further investigate the extent to which altered fecal metabolites are associated with changes in the plasma metabolome in CUMS rats, we analyzed the differential metabolites of the fecal metabolome separating the CUMS rats and the healthy controls at the 4th week of CUMS modeling with OPLS-DA. A total of 32 differential metabolites were selected (variable importance for the projection >1, adjusted *P* value < 0.05), among which 26 metabolites were structurally identified (Supplementary Table 1). Targeted metabolomic profiling further corroborated the differences in abundance between the CUMS rats and the healthy controls (data not shown). To investigate the relationship between the changes in the fecal and plasma metabolomes, PA with Euclidean distances was performed. Results of PA on the differential metabolites of feces and plasma revealed a strong congruency of sample separation (Fig. 3a). To further investigate whether the differential abundance of fecal metabolites correlates with the changes in the plasma metabolome, we carried out CCA. The results of CCA revealed a clear separation between the CUMS-enriched and the control-enriched plasma metabolites/spectral features (Fig. 3b, Supplementary Table 2) along the vectors for L-threonine, isoleucine, alanine, serine, tyrosine, and oxidized proline. These results suggest that these fecal amino acids, which are less abundant in the CUMS rats, contribute to changes in the plasma metabolome in CUMS rats.

### Metabolites with differential abundance are associated with depressive-like phenotypes

**Fig. 4:**
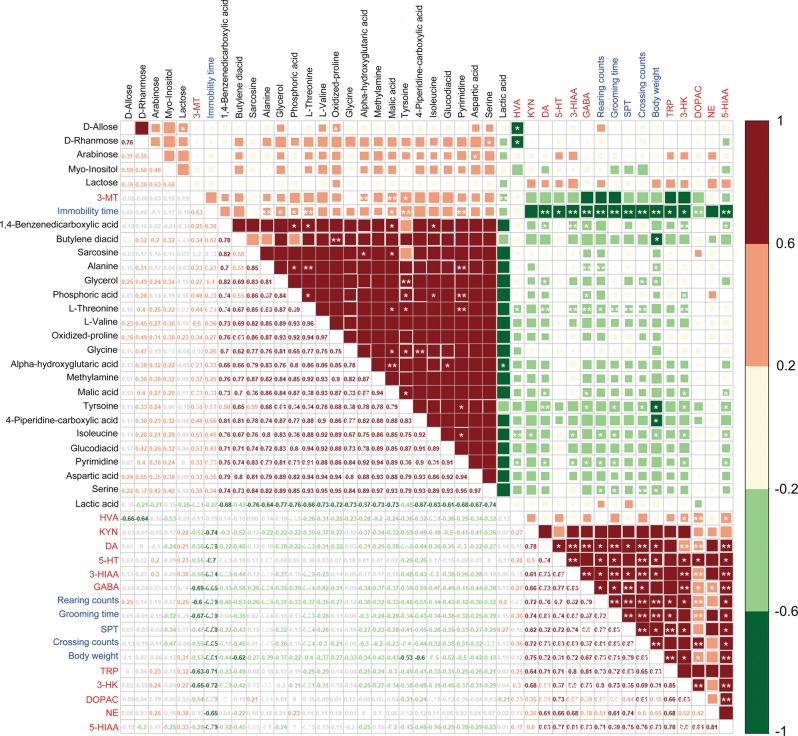
Spearman correlations between the differential fecal metabolites, behavior indices, and neurotransmitters in the hippocampus. Spearman’s rank correlation coefficient among 6 behavior indices, 12 neurotransmitters in the hippocampus, and 26 fecal metabolites that differed significantly in abundance between the CUMS group and the healthy control group (see Supplementary Table 3 for details). Axis label: black, fecal metabolites; red, neurotransmitters; blue, behavior indices. Numbers on the lower left panel: value of correlation coefficient; symbols on the upper right panel: results of significance test; *adjusted *P* < 0.05, **adjusted *P* *<* 0.01. CUMS, chronic unpredictable mild stress

Neurotransmitter depletion in the hippocampus is a key feature of depressive-like symptoms^50^. To study the correlations between the changes in the abundances of fecal metabolites and depressive-like phenotypes, we tested for Spearman correlations among the fecal metabolites, depressive-like behaviors, and neurotransmitters in the hippocampus (Fig. 4). Among the 26 fecal metabolites whose abundances differed in the CUMS rats and the healthy controls, two correlation clusters were observed from the heatmap of the Spearman correlation with hierarchical cluster analysis (Fig. 4). One cluster consisted of oligosaccharides and the other consisted mostly of amino acids. In the oligosaccharide cluster, D-allose, D-rhamnose, and myo-inositol were negatively correlated with homovanillic acid in the hippocampus (Spearman correlation, adjusted *P* value < 0.05; Supplementary Table 3). Within the cluster of amino acids, most metabolites were negatively correlated with changes in neurotransmitters in the hippocampus and with depressive-like behaviors (Fig. 4, Supplementary Table 3).

Notably, among the altered fecal amino acids that were responsible for the CCA separation between the CUMS-enriched and control-enriched plasma metabolomic features (Fig. 3b), fecal tyrosine levels were negatively correlated with body weight; fecal alanine, tyrosine, and isoleucine levels were positively correlated with plasma phosphoric acid; fecal alanine and L-threonine levels were positively correlated with plasma pyrimidine levels; and fecal alanine and tyrosine levels were positively correlated with plasma L-threonine levels (Spearman correlation, adjusted *P* < 0.05; Fig. 4, Supplementary Table 3). These results suggest that the altered fecal metabolites, especially amino acids, were correlated with each other and with the depressive-like phenotypes.

### The fecal microbiota contributes to changes in fecal amino acid metabolisms in CUMS rats

**Fig. 5:**
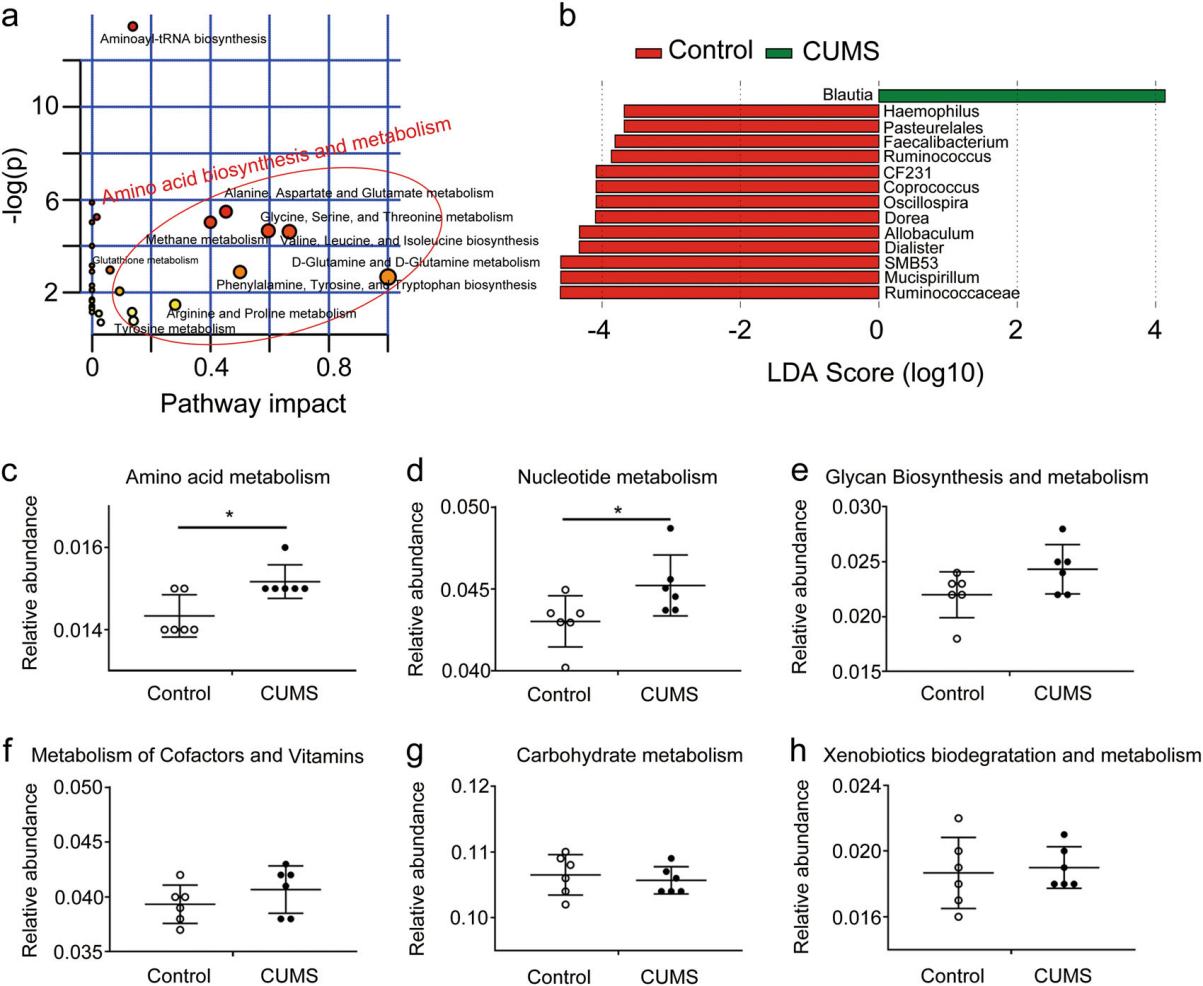
Consistency between the metabolic pathway enrichments of the differential fecal metabolites and metabolic predictions of the altered fecal microbiota. **a** Metabolic pathway-enrichment analyses of the metabolites that were changed in the CUMS rats relative to the healthy controls. The matched metabolic pathways are displayed as circles. The size and color of each circle are based on the pathway impact value and the *P* value, respectively. **b** Histogram of linear discriminant analysis (LDA) scores computed by LEfSe analysis assessing the differentially abundant bacterial taxa between the CUMS rats (green panel) and the healthy controls (red panel). **c**–**h** PICRUSt functional inference of the altered fecal microbiota. Student’s *t* tests were applied to calculate between-group statistical significance, *P *<* 0.05. CUMS, chronic unpredictable mild stress

The fecal metabolome is influenced by several factors, including the host genetic background, the gut microbiota, and environmental elements such as diet. Because the host background and environmental factors are essentially the same for the CUMS rats, we investigated whether the gut microbiota was responsible for the altered fecal metabolites. We observed a statistically significant difference in α-diversity analysis (Supplementary Fig. 4a) and a clear separation in β-diversity analysis (Supplementary Fig. 4b) of fecal microbiota between the CUMS rats and the healthy controls, suggesting that the microbiota composition of CUMS rats is significantly different from that of the healthy controls. Metabolic pathways were first deduced from the differential fecal metabolites using the pathway enrichment element of the MetaboAnalyst web portal. Pathways of amino acid biosynthesis and metabolism were significantly enriched after exposure to CUMS (Fig. 5a). The composition and function of fecal microbiota were then studied by 16s rDNA sequencing. We carried out Linear discriminant analysis Effect Size (LEfSe) analysis to study the differential composition of fecal microbiota in the CUMS rats and the healthy controls. We observed that *Blautia* was the only genus enriched in the CUMS rats, while a total of 13 genera were enriched in the healthy controls (Fig. 5b, Supplementary Table 4). The functional profiles of the altered fecal microbiota of the CUMS rats were predicted by PICRUSt analysis (Fig. 5 c–h). Among the six predicted metabolic pathways, amino acid metabolism and nucleotide metabolism (Fig. 5 c, d) were significantly varied in the fecal microbiota of CUMS compared to those of the healthy controls. The consistency between the enriched metabolic pathway of the fecal metabolome and the predicted functional profile of the fecal microbiota suggests that the fecal microbiota contributes substantially to changes in fecal amino acid metabolism.

### The altered fecal microbiota is associated with depressive-like symptoms

**Fig. 6:**
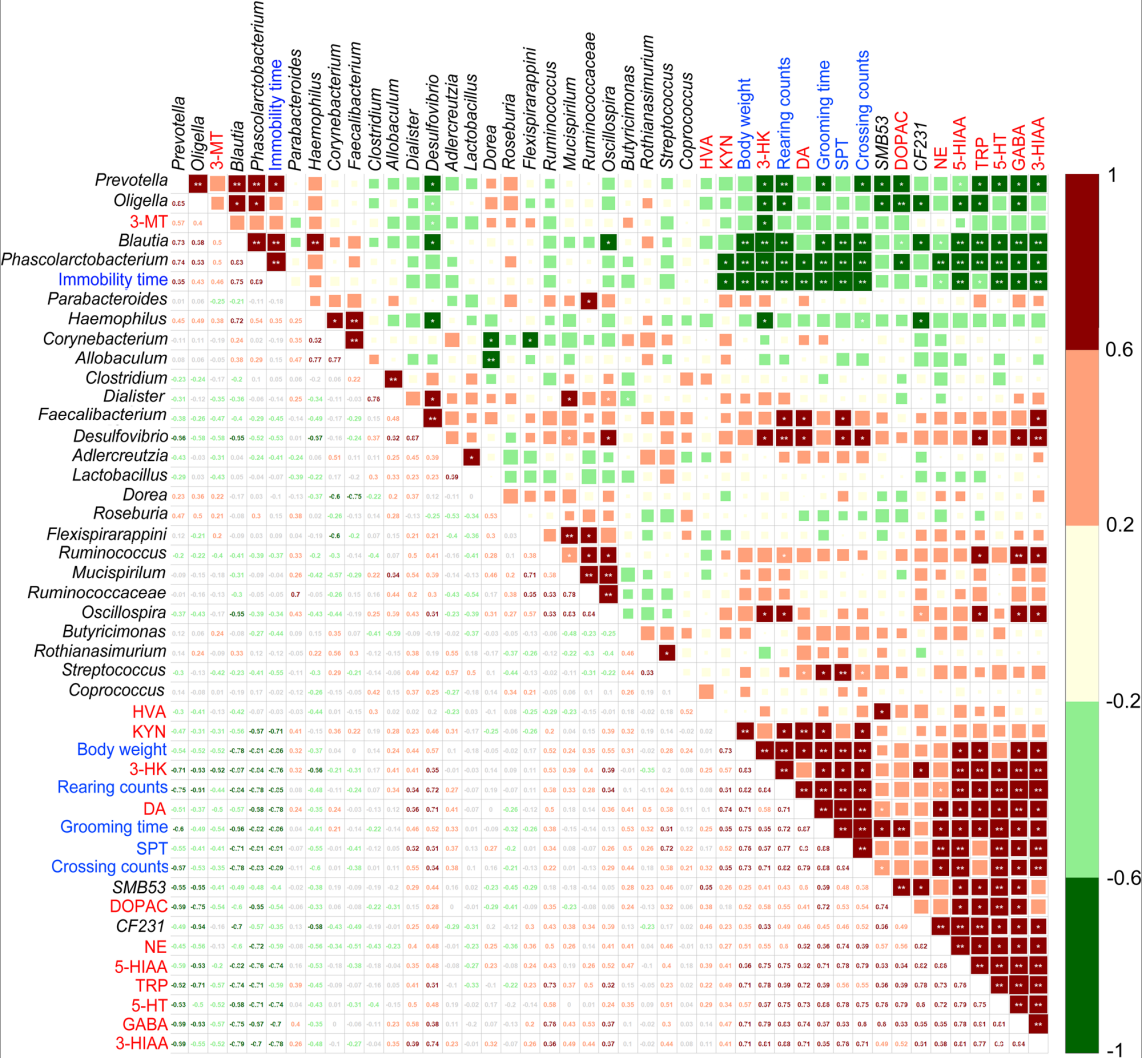
Spearman correlations among the altered fecal microbiota, behavior indices, and neurotransmitters in the hippocampus. Spearman’s rank correlation coefficient among 6 behavior indices, 12 neurotransmitters in hippocampus, and 26 fecal microbiota genera that differed significantly in abundances between the CUMS rats and the healthy controls (see Supplementary Table 5 for details). Axis label: black, fecal microbiota genera; red, neurotransmitters; blue, behavior indices. Number on the lower left panel: value of correlation coefficient; symbols on the upper right panel: values of significance test, *adjusted *P* < 0.05, **adjusted *P* *<* 0.01. CUMS, chronic unpredictable mild stress

To further investigate the extent to which changes in the fecal microbiota are associated with depressive-like symptoms, we performed Spearman rank correlation analysis on differential taxa and behavior indices between the CUMS rats and the healthy controls (Fig. 6). We observed differences in a total of 14 genera. Using hierarchical cluster analysis, we separated the altered fecal microbiota into several clusters. The genera *Prevotella, Oligella, Blautia*, and *Phascolarctobacterium* were positively correlated with each other and with immobility time in the OFT, which was negatively correlated with most of the other behavior indices and most of the neurotransmitters in the hippocampus (adjusted *P* < 0.05; Supplementary Table 5). On the other hand, the genera *Faecalibacterium* and *Desulfovibrio* were positively correlated with neurotransmitter levels in the hippocampus and with most of the behavior indices except the immobility time of OFT (adjusted *P* < 0.05; Supplementary Table 5). The genera SMB53 and CF231 were positively correlated with neurotransmitter levels in the hippocampus and negatively correlated with the genera *Prevotella, Oligella, Blautia*, and *Phascolarctobacterium* (adjusted *P* < 0.05; Supplementary Table 5). In combination with the consistency between the altered fecal metabolome and the altered functional profile of the fecal microbiota and the correlation between the fecal and plasma metabolome, these results led us to conclude that the altered gut microbiota affects the metabolism of amino acids in the host circulating system and is associated with behavior indices of depression.

## Discussion

In this study, we applied an integrative analysis of the fecal and plasma metabolomes to reveal that the changes in amino acid abundances in the fecal metabolome significantly contribute to changes in the plasma metabolome and are associated with the depressive-like phenotypes in CUMS rats. Our results reveal the consistency of perturbed amino acid metabolism between the pathway enrichment of the altered fecal metabolome and the predicted functional profile of the altered fecal microbiota in the CUMS rats. The findings of this study suggest that changes in the metabolites of the gut microbiota play an important role in the pathogenesis of depressive-like behaviors.

Since the establishment of extensive connections between the gut microbiota and disease, there has been great interest in the mechanisms that underlie this correlation^51–53^. Metabolites have been shown to be the key messengers in the bi-directional communication between the gut microbiota and the host^54,55^. Although depression has been correlated with metabolic changes in the circulating system^56^ and the central nervous system^57^, the contribution of the gut microbiota to these changes is not yet fully understood. In this study, changes in the fecal metabolome were found to arise before changes in the plasma metabolome of CUMS rats during the development of depressive-like behaviors. Furthermore, the changes in the abundances of several amino acids in the feces of CUMS rats, including L-threonine, isoleucine, alanine, serine, tyrosine, and oxidized proline, were found to be associated with the changes in the plasma metabolome. Disorders of amino acid metabolism have been associated with the pathophysiology of depression^31,58^. Perturbations in amino acid metabolism have been reported to occur in the prefrontal cortex of the learned helplessness rat model of depression^57^. Glutamine deficiency in the prefrontal cortex increases depressive-like behaviors in male mice, and a direct infusion of L-glutamine reversed the increased immobility in forced swim test^59^. In addition, plasma levels of alanine and L-serine were reported to be correlated with the severity of depression^56^. Consistent with these previous reports, we observed substantially different levels of the above-mentioned amino acids in the feces and plasma of CUMS rats with depressive-like behaviors. We also observed significant correlations between the abundances of these amino acids and depressive-like phenotypes. Taken together, the findings from this study and these previous reports suggest that disorders of amino acid metabolism in the gut play a crucial role in the pathophysiology of depression.

Previous works indicate that the gut microbiota plays an important role in the pathophysiology of depression^60,61^, although the pathways linking gut bacteria with the central nervous system are not fully elucidated^19,61–63^. Consistent with our observation of the differential enrichment of bacterial genera between CUMS rats and healthy controls, the genus *Blautia* was reported to be more abundant in the microbiota of patients with MDD^64^. The relative abundances of taxa *Coprococcus* and *Dorea* were previously shown to be reduced in mice exposed to a prolonged stressor^65^, and the abundances of taxa *Allobaculum* and *Mucispirillum* were reduced in mice exposed to sub-chronic and mild social defeat stress^66^. Our findings are consistent with prior observations that the altered gut microbiota is closely correlated with depression.

Associations between depression and changes in the metabolic pathways of these amino acids have been previously reported. Changes in alanine, aspartate, and glutamate metabolism have been reported to be associated with the antidepressant effects of venlafaxine^67^. Changes in glycine, serine, and threonine metabolism have been reported to be associated with treatment-resistant depression, and a 5-week course of antidepressant treatment modulates the serum levels of these excitatory amino acids^68^. Finally, the biosynthesis of valine, leucine, and isoleucine is believed to play a crucial role in the development of depression, possibly through the activation of the mammalian target of rapamycin pathway. These three branched-chain amino acids could therefore serve as biomarkers of depression^68^. Abnormalities in the glutamine–glutamate cycle, which is essential for the communication between glia and neurons, have been reported to be critical in the pathophysiology of depression^69^, and decreased glutamate/glutamine levels were shown to mediate cytidine’s efficacy in treating bipolar depression^70^. The inflammation-induced metabolism of phenylalanine, tyrosine, and tryptophan is thought to serve as for monitoring the progression of depression and for guiding timely psychiatric interventions^71^. Our findings, together with previous reports, demonstrate that changes in amino acid metabolism in the gut microbiota contribute directly to plasma metabolome disorder and are associated with depressive-like phenotypes. Further studies are required to elucidate the mechanisms by which these amino acids contribute to the pathophysiology of depression.

In summary, our results demonstrate that the gut metabolome contributes markedly to any disorder in the circulating metabolome of CUMS rats and is associated with depressive-like behaviors. These findings suggest that an altered gut metabolome may serve as a promising target for novel antidepressants.

## Supplementary information

Supplementary figure legends

Supplementary Figure 1

Supplementary Figure 2

Supplementary Figure 3

Supplementary Figure 4

Supplementary Table 1

Supplementary Table 2

Supplementary Table 3

Supplementary Table 4

Supplementary Table 5

## Data Availability

The raw 16s rDNA sequencing data have been deposited in the GenBank Sequence Read Archive with the BioProject ID PRJNA485720. Codes for CIA, CCA, PA, Spearman correlation, and PERMANOVA analysis can be found in the help file of each R package. Default settings were used unless otherwise mentioned.
