# Correction: Altered gut metabolome contributes to depression-like behaviors in rats exposed to chronic unpredictable mild stress

**DOI:** 10.1038/s41398-019-0645-9

**Published:** 2019-11-07

**Authors:** Li Jianguo, Jia Xueyang, Wang Cui, Wu Changxin, Qin Xuemei

**Affiliations:** 1 Laboratory of Microbiome and Health, Institutes of Biomedical Sciences, Shanxi University, Taiyuan, 030006 China; 2 Key Laboratory of Chemical Biology and Molecular Engineering of Ministry of Education, Shanxi University, Taiyuan, 030006 China; 3 Modern Research Center for Traditional Chinese Medicine, Shanxi University, Taiyuan, 030006 China

**Keywords:** Depression, Physiology

**Correction to:Translational Psychiatry**

10.1038/s41398-019-0391-z published online January 2019

After publication, the author’s noted that the original Article contained clerical errors in the Results section.

The following passage from the original text:

“For example, we observed significant differences in body weight (Fig. 1c), most OFT indices (time spent immobile (Fig. 1d)), crossing counts (Fig. 1e), and rearing counts (Fig. 1f) starting in the 3rd week of exposure to CUMS. We did not observe significant changes in sucrose preference rate (Fig. 1b) or grooming time of OFT (Fig. 1g) until the last week of CUMS modeling, although an isolated notable change was evident in the 1st week.”

Has been updated to:

“For example, we observed significant differences in body weight (Fig. 1c), two OFT indices (time spent immobile (Fig. 1d)), and rearing counts (Fig. 1f) starting in the 3rd week of exposure to CUMS. We did not observe significant changes in sucrose preference rate (Fig. 1b), grooming time (Fig. 1e) or crossing counts (Fig. 1g) of OFT until the last week of CUMS modeling, although an isolated notable change was evident in grooming time (Fig. 1e) at the 1st week.”

