# Supplementary material for: Altered gut metabolome contributes to depression-like behaviors in rats exposed to chronic unpredictable mild stress
Source: Transl Psychiatry. 2019 Jan 29;9:40. doi: 10.1038/s41398-019-0391-z (PMC6351597; doi:10.1038/s41398-019-0391-z)
Supplement: Supplementary file 1 — Supplementary figure legends [file 41398_2019_391_MOESM1_ESM.doc]

**Suppl. Fig. 1 Behavior tests for evaluation of the CUMS modeling.** To evaluate the CUMS modeling, behavior tests were performed after four-week CUMS exposure. a, sugar preference test. b, time spent immobile in open field test. c, time spent immobile in forced swimming test. d, time spent immobile in tail suspension test. Student’s t-tests were applied to calculate between-group statistical significance, **** *P* < 0.0001.

**Suppl Fig. 2 Principle component analyses of hippocampus and prefrontal cortex metabolomes between CUMS rats and healthy controls. Principle component analyses (**PCAs) were performed to compare the between-group variations in metabolome data from hippocampus (a) and prefrontal cortex (b) samples collected after fourth-week CUMS exposure. The first two axes (PC1 & PC2) were selected for the score plots. Purple dots: rats of the CUMS group; blue dots: rats of the healthy control group.

**Suppl Fig. 3 Principal component analysis and Co-Inertia analysis of plasma and fecal metabolome data obtained at five time points of the CUMS modelling.** Principal component analyses (PCAs) were performed to compare the between-group variations on metabolome data from samples obtained before CUMS modeling (W0), and at every week of CUMS modeling (W1-W4). The first two axes (PC1 & PC2) were selected for the score plots. Red dots: rats of the CUMS group; black dots: rats of the healthy control group. a1-a5: plasma metabolome, b1-b5: fecal metabolome. Co-Inertia (CIAs) were performed with R package omicade4 on metabolomic data obtained from the five time points (W0-W4) of CUMS modeling (c1-c5). The dots represent metabolites or unidentified features of the metabolomic data (Black: features of the fecal metabolome, Red: features of the plasma metabolome).

**Suppl Fig. 4 Within-group diversity and between-group diversity of the 16S rRNA sequencing results between the CUMS rats and healthy controls.** *α*-diversity (a) was performed by R package Phyloseq, *β*-diversity (b) was performed by QIIME pipeline (v1.8.0) to determine the differences of microbial composition between CUMS rats and healthy controls. ** *P* < 0.01.
