# Supplementary figures and images for: Altered gut metabolome contributes to depression-like behaviors in rats exposed to chronic unpredictable mild stress
Source: Transl Psychiatry. 2019 Jan 29;9:40. doi: 10.1038/s41398-019-0391-z (PMC6351597; doi:10.1038/s41398-019-0391-z)

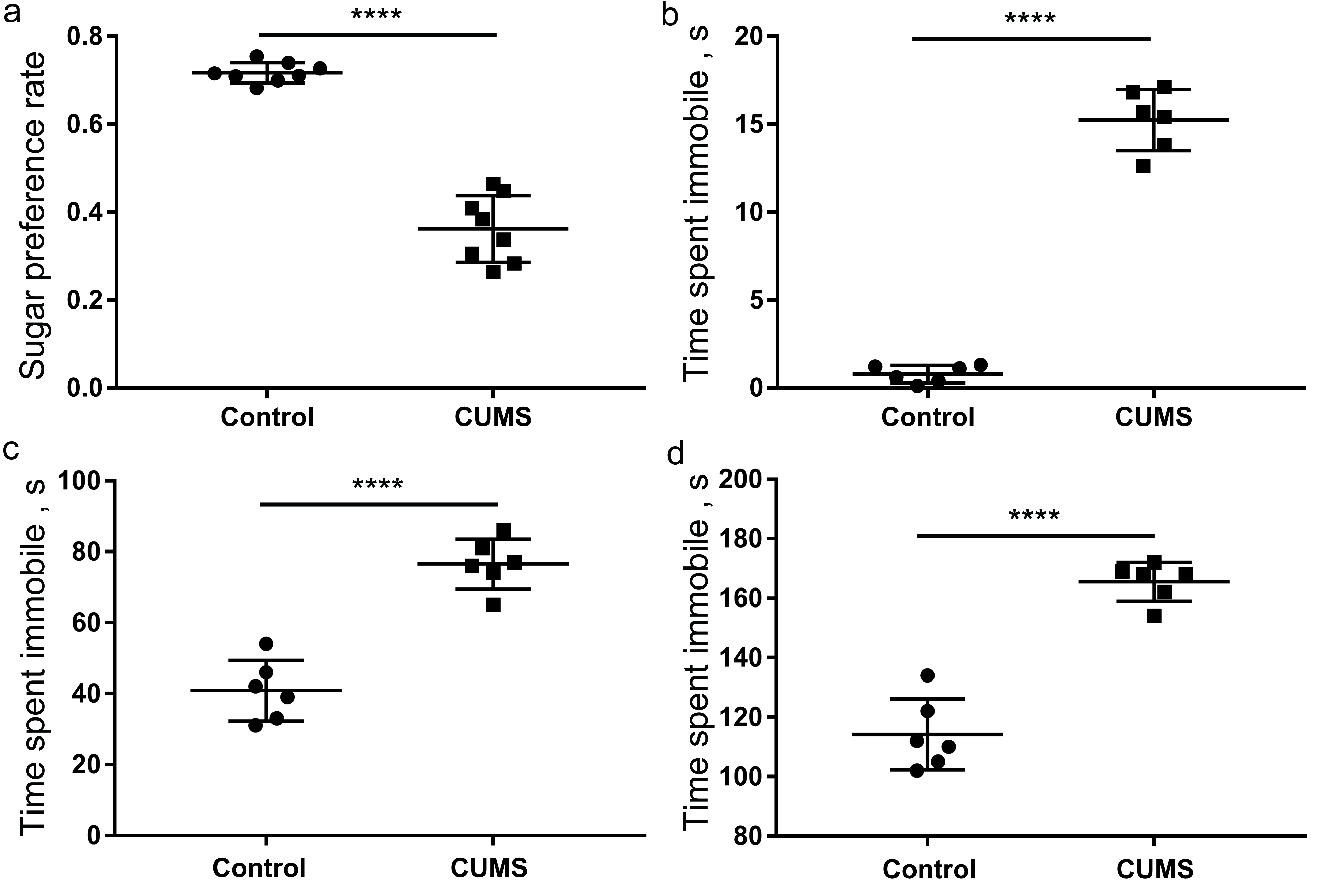

Supplement: Supplementary file 2 — Supplementary Figure 1 [file 41398_2019_391_MOESM2_ESM.tif]

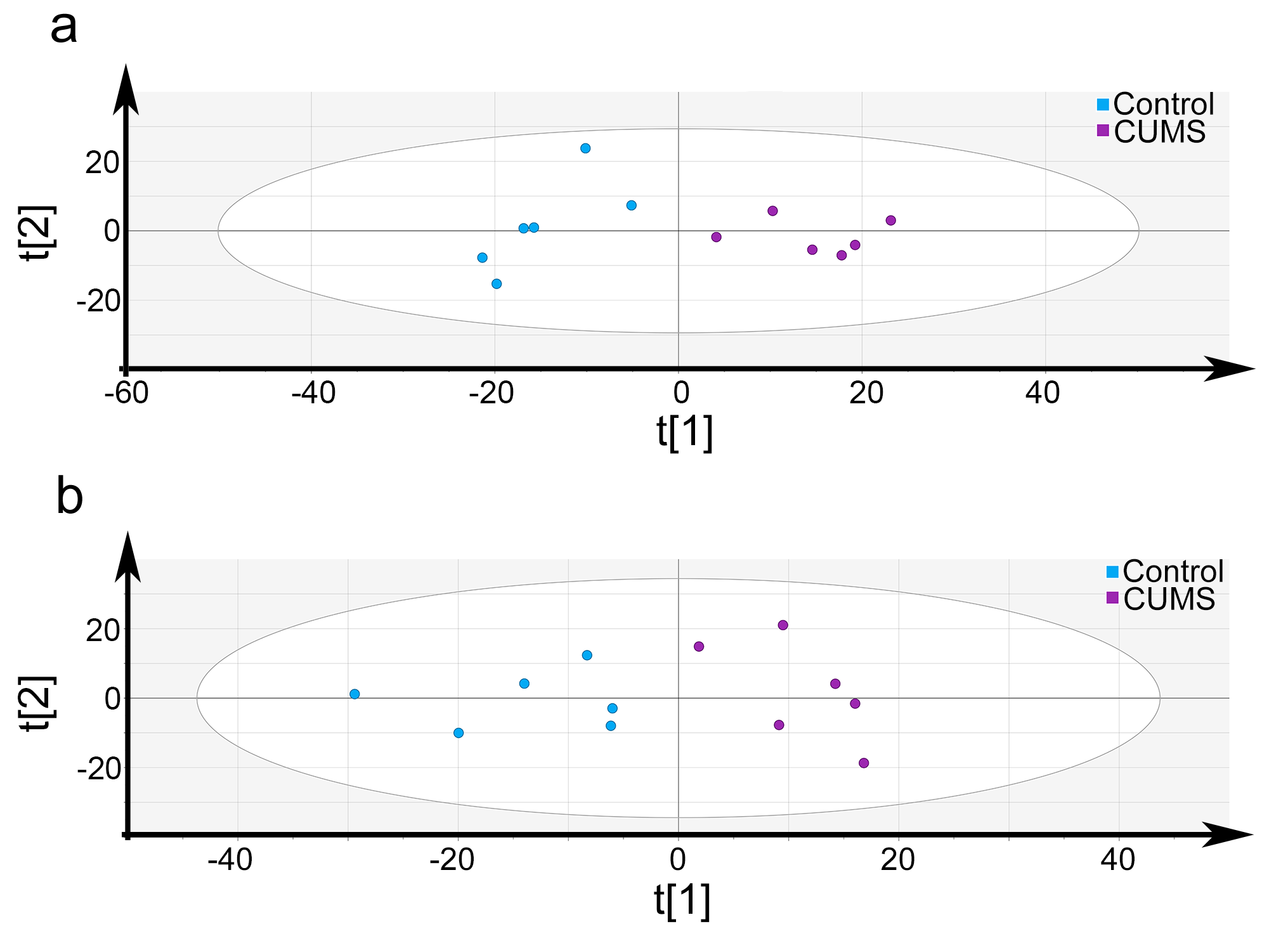

Supplement: Supplementary file 3 — Supplementary Figure 2 [file 41398_2019_391_MOESM3_ESM.tif]

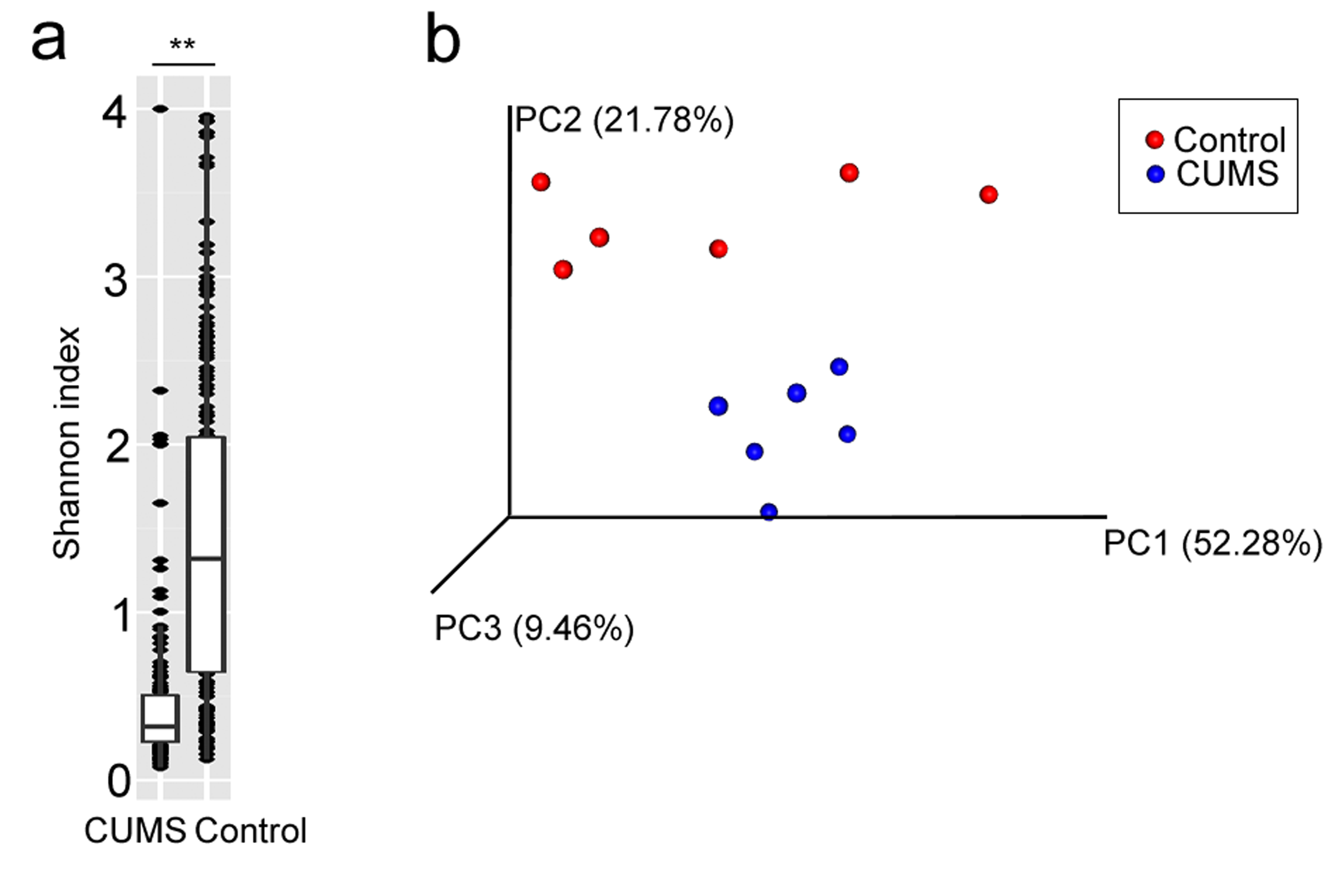

Supplement: Supplementary file 5 — Supplementary Figure 4 [file 41398_2019_391_MOESM5_ESM.tif]
